# Metabolic profiling of galectin-1 and galectin-3: a cross-sectional, multi-omics, association study

**DOI:** 10.1038/s41366-024-01543-1

**Published:** 2024-05-22

**Authors:** Emanuel Fryk, Vagner Ramon Rodrigues Silva, Lena Strindberg, Robin Strand, Håkan Ahlström, Karl Michaëlsson, Joel Kullberg, Lars Lind, Per-Anders Jansson

**Affiliations:** 1 Department of Molecular and Clinical Medicine, Institute of Medicine, Sahlgrenska Academy, University of Gothenburg, Gothenburg, Sweden; 2 Department of Information Technology, Uppsala University, Uppsala, Sweden; 3 Division of Radiology, Department of Surgical Sciences, Uppsala University, Uppsala, Sweden; 4 Antaros Medical AB, BioVenture Hub, Mölndal, Sweden; 5 Department of Surgical Sciences, Medical Epidemiology, Uppsala University, Uppsala, Sweden; 6 Department of Medical Sciences, Uppsala University, Uppsala, Sweden

**Keywords:** Obesity, Type 2 diabetes

## Abstract

**Objectives:**

Experimental studies indicate a role for galectin-1 and galectin-3 in metabolic disease, but clinical evidence from larger populations is limited.

**Methods:**

We measured circulating levels of galectin-1 and galectin-3 in the *Prospective investigation of Obesity, Energy and Metabolism* (POEM) study, participants (*n* = 502, all aged 50 years) and characterized the individual association profiles with metabolic markers, including clinical measures, metabolomics, adipose tissue distribution (Imiomics) and proteomics.

**Results:**

Galectin-1 and galectin-3 were associated with fatty acids, lipoproteins and triglycerides including lipid measurements in the metabolomics analysis adjusted for body mass index (BMI). Galectin-1 was associated with several measurements of adiposity, insulin secretion and insulin sensitivity, while galectin-3 was associated with triglyceride-glucose index (TyG) and fasting insulin levels. Both galectins were associated with inflammatory pathways and fatty acid binding protein (FABP)4 and -5-regulated triglyceride metabolic pathways. Galectin-1 was also associated with several proteins related to adipose tissue differentiation.

**Conclusions:**

The association profiles for galectin-1 and galectin-3 indicate overlapping metabolic effects in humans, while the distinctly different associations seen with fat mass, fat distribution, and adipose tissue differentiation markers may suggest a functional role of galectin-1 in obesity.

## Introduction

There is increasing interest in the role of galectins in human health [1–3]. Highly conserved between species, galectins mediate a range of physiological effects, including inflammation, tissue remodeling, and metabolism [4]. Galectins miss the classical signal peptide for secretion, are found in high intracellular concentrations, but are readily measurable in human blood [5]. Furthermore, galectins are known to bind to numerous glycoproteins and glycolipids on the cell surface, but the definitive cellular origin, and physiological role of circulating galectins has not yet been demonstrated [5].

Meta-analyses have reported elevated levels of circulating galectin-3 in individuals with obstructive sleep apnea [6], diabetic nephropathy [7] and high plasma levels are associated with increased risk of cardiovascular mortality [8]. Plasma galectin-3 is also higher during early pregnancy in women with gestational diabetes, suggesting a role in metabolic disease [9]. Galectin-3 is involved in the development of insulin resistance through direct interactions with the insulin receptor, with altered insulin-regulated glucose metabolism in adipocytes, myocytes and hepatocytes [10]. Galectin-3 has also been related to lipid content in the liver through interactions with the lipid uptake marker CD36 and PPAR-γ, and ablation of galectin-3 in mice provided a protective effect on non-alcoholic steatohepatitis by reducing liver steatosis [11, 12].

Emerging evidence suggests that another lectin, galectin-1, could have an equal or even more significant role in obesity and metabolic disease [13–16]. An unbiased proteomic analysis of adipose tissue interstitial fluid in patients with type 2 diabetes (T2D) and healthy controls reported elevated galectin-1 levels in T2D [14]. A cross-sectional community-based study of 989 individuals, found that serum galectin-1 was independently associated with high BMI and serum insulin levels [13]. Increased serum galectin-1 levels were also associated with a higher incidence of T2D in a longitudinal study of 4022 individuals [15]. Furthermore, it has been shown that Lgals1^−/−^ mice on high-fat diet gain less weight than wild-type mice and that galectin-1 interacts with PPAR-γ signaling [16]. Mechanistic studies also suggest a direct role for galectin-1 on lipid metabolism in cultured adipocytes [17]. Taken together, it is likely that the metabolic effects of galectin-1 found in animal models, explain the associations between galectin-1 and T2D in humans.

With the growing number of reports on metabolic effects of different galectins, and the discovery of feasible galectin-1 and galectin-3 inhibitors for human treatment, larger studies on human metabolism are needed [1, 3]. There is also a need for context and overview as to which effects are specific for each galectin, and which effects are general for many galectins [18]. Here, we seek to assess the association profiles of galectin-1 and galectin-3 to glucose homeostasis and different obesity-related variables in the cross-sectional, population-based *Prospective investigation of Obesity, Energy and Metabolism* (POEM) cohort study from Sweden.

## Subjects and methods

### Design

This study adopts a cross-sectional, population-based association study design in a cohort (*n* = 502) of mainly white participants from urban Uppsala, Sweden (Supplementary Fig.).

### POEM study participants

Clinical characteristics and other details of the POEM cohort have been published previously [19–21] but is also shown in a Supplementary Table. In brief, 502 participants were invited to participate 1 month after their 50th birthday. Weight, height, waist, and hip measurements were recorded, and total body fat was assessed through bio-impedance (Tanita BC-418, Tokyo, Japan). Participants performed an oral glucose tolerance test (OGTT), with ingestion of 75 g of glucose and continuous measurements of glucose and insulin every 30 min for 2 h. The homeostatic model assessment (HOMA), and the Matsuda index were calculated as previously defined [22, 23]. Further, we calculated TyG index [24] known to be associated with insulin resistance and increased risk to develop cardiometabolic disease [24–26].

### Blood analysis

Blood was collected after overnight fasting. Analyses were conducted using standard laboratory procedures. Plasma C-peptide levels were measured by enzyme-linked immunosorbent assay (ELISA, Mercodia, Uppsala, Sweden, within assay variation CV < 5%). Plasma galectin-1 levels were determined using ELISA (intra- and inter-assay coefficients of variation were 7.1% and 9.5%, respectively, R&D systems, USA), while plasma galectin-3 was measured with the proximity extension assay technology (Olink, Sweden) [27]. Galectin measurements were performed on all volunteers but one, due to a missing sample.

### Imaging and imiomics analyses

A dual-energy X-ray absorptiometry scanner (DXA; Lunar Prodigy, GE Healthcare, USA) determined the participants’ regional (trunk, leg, arm) and total body fat and lean mass. The precision error was 1.5% and 1.0% for total fat and lean mass, respectively. The validity of fat mass derived by Lunar Prodigy has been evaluated against the 4-compartment model, the tool that is currently considered the gold-standard method of body composition appraisal, resulting in 1.7–2.0% higher fat mass estimates with this narrow fan-beam DXA equipment [28].

Whole-body magnetic resonance imaging (MRI) was conducted using a 1.5 T clinical MR system (Philips Achieva, Philips Healthcare, Best, Netherlands). The Imiomics technique utilized in POEM has been described in detail previously [29]. In the Imiomics-analysis, the participants’ voxel-based MRI imaging data are deformed to fit a reference image. The resulting deformation data allow for voxel-wise whole-body statistical analysis of tissue volumes and fat content from the imaging results in relation to non-imaging variables [29, 30]. Quantifying the visceral adipose tissue (VAT) and subcutaneous adipose tissue (SAT) depots was performed by deforming manually defined depots to all subjects. The quantification of liver fat and pancreas fat was performed using dedicated imaging and analysis protocols [30, 31].

### Nightingale metabolomics analysis

Targeted metabolomics measurements were performed on stored fasting plasma samples using the Nightingale proton nuclear magnetic resonance (NMR) metabolomics platform (Nightingale Health Ltd., Helsinki, Finland). The method has previously been described in detail, and utilized in several large epidemiological studies [32–34]. In brief, a total of 224 metabolites are measured through this technique from the NMR-spectral analysis of two molecular windows, covering various aspects of an individual’s metabolic profile; 171 lipoprotein-related variables and 53 variables related to other metabolic classes are included in the full analysis (https://research.nightingalehealth.com/, last accessed on 2022-05-18).

In this project, we focused on estimates from the main metabolic pathways, including total VLDL (very low-density lipoprotein), LDL (low-density lipoprotein), and HDL (high-density lipoprotein) levels of cholesterol and triglycerides, and total fatty acid levels as well as levels separated by saturation status, amino acids, and other non-lipid metabolites. Stratified data on different lipoprotein particle sizes and markers not directly related to nutrient metabolism, including creatinine and albumin, were excluded in this analysis to simplify the presentation of results.

### Metabolon metabolomics analysis

Non-targeted metabolomics measurements were performed on stored fasting plasma samples as previously detailed (Metabolon Inc., USA) [21]. In brief, metabolomics analyses include combining measurements from four different analyses on each sample: two reverse phases (RP)/UPLC-MS/MS (ultra-performance liquid chromatography-mass spectroscopy/MS) methods with positive ion mode electrospray ionization (ESI), one RP/UPLC-MS/MS method with negative ion mode ESI, and one hydrophilic interaction liquid chromatography (HILIC)/UPLC-MS/MS method with negative ion mode ESI. Only annotated, non-xenobiotic metabolites with a call rate >75% were used in the analyses, and values were normalized and given in arbitrary units. Linear regression models assessed the association between each metabolite and galectin-1 or -3 levels, adjusted for sex, education, smoking, exercise habits, and BMI. All Human Metabolome Database IDs of metabolites with a statistically significant association for each galectin, after Bonferroni adjustment for the total number of analyzed metabolites, were uploaded to the MetaboAnalyst 5.0 website in order to perform a targeted metabolic pathway analysis (https://www.metaboanalyst.ca/, accessed on 2023-02-23).

### Proteomic analysis

Utilizing proximity extension assay technology coupled with next-generation sequencing (NGS) (Olink Proteomics, Uppsala, Sweden), 1320 proteins from the human proteome were measured in fasting plasma samples [35]. In brief, two oligonucleotide-coupled antibodies are used for each protein. The oligonucleotides hybridize to form a DNA template for NGS, when the antibodies bind close enough on the target. This allows for precise quantification of the proteins. Linear regression models determined the association between each protein and galectin-1 or -3, adjusted for sex, education, smoking, exercise habits, and BMI. All proteins with a statistically significant association for each galectin, after Bonferroni adjustment for the total number of analyzed proteins, were uploaded to the Reactome pathway analysis tool (https://reactome.org/, accessed on 2023-01-27) to explore possible signaling pathways associated with each galectin [36].

### Statistics

Continuous variables were z-transformed to allow for effect size comparisons between galectin-1 and galectin-3, with the average set to 0 and the standard deviation to 1. Skewed variables were log-transformed with the natural logarithm for a near normal distribution as indicated in tables. Data were analyzed using linear models, adjusted for sex, education, smoking, and exercise habits unless otherwise specified. The confounders were chosen as plausible factors to influence metabolic outcomes. Due to the previously reported association between galectin-1 and BMI, additional models also adjusting for BMI are presented. A *p* value below 0.05 was considered statistically significant. Only proteins and metabolites statistically associated with the galectins after Bonferroni adjustment for multiple comparisons were included in the subsequent exploratory pathway analysis.

## Results

### The association profile for galectin-1 and galectin-3 with adipose tissue variables

Galectin-1 levels were associated with all measures of adiposity in the analysis. The highest β-coefficients were seen with BMI, VAT, and SAT (Table 1). Associations were also significant for fat deposition in sub-compartments from the liver and pancreas, and to total fat-free mass. After additional adjustment for BMI, the galectin-1 associations with fat mass, VAT and SAT were still significant. Plasma galectin-3 levels did not present statistically significant linear associations with any body composition variable.

**Table 1:** Association between galectin-1 or galectin-3 levels and metabolic variables in the POEM study cohort.

	Galectin-1				Galectin-3			
	*β* (CI)	*p* value	BMI-adjusted *β* (CI)	Adj. *p* value	*β* (CI)	*p* value	BMI-adjusted *β* (CI)	Adj. *p* value
Body composition								
Weight	0.30 (0.23: 0.37)	**<0.001**	0.02 (−0.02: 0.07)	0.260	0.04 (−0.03: 0.12)	0.278	−0.01 (−0.05: 0.02)	0.483
Body mass index	0.37 (0.29: 0.45)	**<0.001**	–	–	0.07 (−0.01: 0.16)	0.095	–	–
Fat mass	0.27 (0.21: 0.33)	**<0.001**	0.05 (0.01: 0.09)	**0.008**	0.05 (−0.01: 0.12)	0.097	0.01 (−0.03: 0.04)	0.654
Waist-to-hip ratio	0.13 (0.05: 0.21)	**0.002**	−0.02 (−0.10: 0.06)	0.572	0.05 (−0.03: 0.13)	0.209	0.02 (−0.05: 0.09)	0.552
VAT^a^	0.35 (0.26: 0.45)	**<0.001**	0.17 (0.10: 0.25)	**<0.001**	0.06 (−0.04: 0.15)	0.265	0.03 (−0.04: 0.11)	0.339
SAT^a^	0.38 (0.28: 0.47)	**<0.001**	0.15 (0.08: 0.21)	**<0.001**	0.04 (−0.06: 0.14)	0.404	0.02 (−0.04: 0.08)	0.563
Liver fat^a^	0.25 (0.14: 0.35)	**<0.001**	0.06 (−0.04: 0.16)	0.216	0.01 (−0.09: 0.12)	0.787	−0.02 (−0.11: 0.07)	0.656
Pancreas fat^a^	0.13 (0.01: 0.25)	**0.031**	−0.02 (−0.13: 0.10)	0.737	0.07 (−0.04: 0.19)	0.211	0.06 (−0.04: 0.16)	0.264
Lean mass	0.14 (0.09: 0.18)	**<0.001**	−0.01 (−0.05: 0.03)	0.768	0.01 (−0.04: 0.06)	0.66	−0.02 (−0.05: 0.02)	0.358
Glucose metabolism								
fB-Glucose	−0.13 (−0.22: −0.04)	**0.005**	−0.20 (−0.30: −0.10)	**<0.001**	−0.13 (−0.21: −0.04)	**0.006**	−0.13 (−0.22: −0.04)	**0.003**
B-Glucose_120_	0.02 (−0.07: 0.11)	0.650	−0.06 (−0.15: 0.04)	0.254	−0.07 (−0.15: 0.02)	0.123	−0.08 (−0.17: 0.00)	0.065
fS-C-peptide	0.25 (0.17: 0.34)	**<0.001**	0.09 (0.00: 0.17)	**0.041**	−0.03 (−0.12: 0.05)	0.463	−0.07 (−0.14: 0.01)	0.087
fS-Insulin	0.10 (0.01: 0.19)	**0.025**	0.05 (−0.05: 0.15)	0.310	0.12 (0.03: 0.21)	**0.008**	0.11 (0.02: 0.19)	**0.015**
HOMA^a^	0.19 (0.10: 0.27)	**<0.001**	0.05 (−0.04: 0.13)	0.307	0.05 (−0.04: 0.13)	0.300	0.02 (−0.06: 0.10)	0.663
TyG-index	0.21 (0.12: 0.29)	**<0.001**	0.10 (0.01: 0.18)	**0.024**	0.14 (0.06: 0.22)	**<0.001**	0.12 (0.04: 0.019)	**0.004**
Matsuda index^a^	−0.25 (−0.34: −0.17)	**<0.001**	−0.09 (−0.17: −0.01)	**0.035**	−0.05 (−0.14: 0.03)	0.237	−0.02 (−0.09: 0.06)	0.636

All analyses were made on z-transformed variables.

*HOMA* homeostatic model assessment, *SAT* subcutaneous adipose tissue, *VAT* visceral adipose tissue, *TyG* triglyceride-glucose index.

^a^Data were log-transformed using the natural logarithm.

Bold values indicates statistical significant *p* values (*p* < 0.05).

### The association profile for galectin-1 and galectin-3 on metabolic variables

Galectin-1 levels were negatively associated with glucose and positively associated with C-peptide levels, fasting insulin, and insulin resistance measured as HOMA and the Matsuda index (Table 1). Additional adjustment for BMI mitigated most associations. While associations between C-peptide levels, TyG-index and the Matsuda index remained statistically significant, levels of fasting insulin and HOMA results did not. The inverse association with fasting glucose increased both in effect size and degree of significance (lower *p* value). Galectin-3 levels were also negatively associated with glucose levels and positively associated with fasting insulin levels and TyG-index. However, galectin-3 was not associated with levels of C-peptide or insulin resistance as measured by HOMA and the Matsuda index. Adjustments for BMI drastically weakened the association with fasting insulin, while increasing the inverse association with fasting glucose levels, as observed for galectin-1.

### Imiomics associations for galectin-1 and galectin-3

**Fig. 1:**
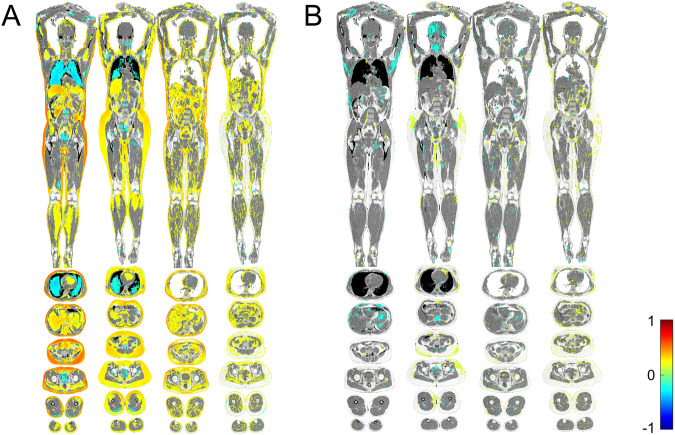
Imiomics associations with galectin-1 and galectin-3 in male and female participants. Imiomics associations with galectin-1 (**A**) and galectin-3 (**B**) in one representative male (left) and female (right) participant. Associations are presented for tissue volume (left) and fat content (right). Significant (*p* < 0.05) voxel wise non-parametric Spearman rank coefficient correlation values (r-maps) are shown in the color scale. Positive associations are shown in warm colors (yellow—red) and negative associations are shown in cool colors (green—blue), see the color bars. Pixels with non-significant correlations show the underlying water signal values (gray scale).

Galectin-1 levels were related to SAT volume in both the upper and lower parts of the body. These associations were generally more pronounced in men than women (Fig. 1A). Galectin-1 was furthermore related to the size of the liver and VAT, and to skeletal muscle volume in the legs. Again, these relationships were more pronounced in men. Levels of galectin-1 were also related to heart size and inversely related to the size of the lungs in both sexes. The fat fraction of SAT and in the liver was related to galectin-1 levels, especially in men. Galectin-1 levels were furthermore related to the intramuscular fat fraction in leg skeletal muscle, as well as to pericardial fat fraction in both sexes. Galectin-3 levels were weakly related to the SAT volume in the hip region in women only (Fig. 1B).

### Nightingale metabolomics associations for galectin-1 and galectin-3

**Fig. 2:**
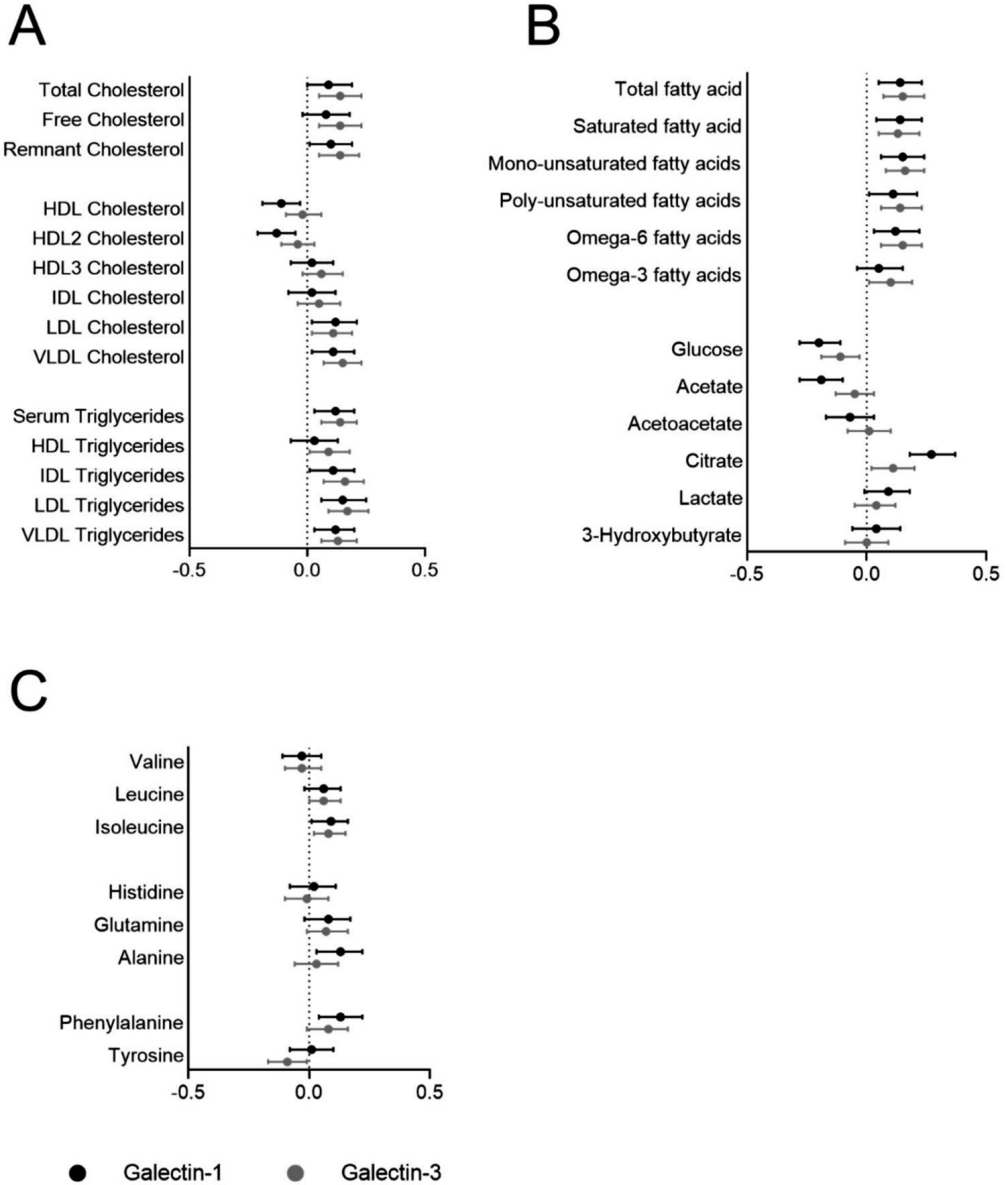
Metabolomic profiling of galectin-1 (black) and galectin-3 (gray). Body mass index-adjusted linear associations for the two markers with markers of lipoprotein metabolism (**A**), fatty-acid and glucose metabolism (**B**), and amino acids (**C**). HDL high-density lipoprotein, IDL intermediate-density lipoprotein, LDL low-density lipoprotein, VLDL very low-density lipoprotein.

The BMI-adjusted associations for galectin-1 and galectin-3 with lipoprotein metabolic markers are presented in Fig. 2A. The two galectins had almost identical association patterns with all markers of lipoprotein metabolism. Both galectins were associated with higher levels of total, remnant, LDL, and VLDL cholesterol, as well as serum triglycerides and triglycerides in most lipoproteins. Although similar effect sizes, only galectin-3 and not galectin-1 levels were statistically significantly associated with free cholesterol and HDL triglyceride levels. Conversely, there was a statistically significant inverse association between galectin-1 and HDL and HDL2 cholesterol levels, which was not found with galectin-3. Examining the associations with free fatty acid levels and with other metabolites in glucose and fatty acid metabolism (Fig. 2B), the overall similarities between the two proteins were evident. Both galectins were associated with total-, saturated- and unsaturated fatty acid levels. Inverse associations with glucose, and positive associations with citrate, were also seen for both galectin-1 and -3. There was a statistical difference for docosahexaenoic acid which was only significantly associated with galectin-3 levels, and an association with acetate only significant for galectin-1. However, the directions of effect were similar for these markers. Similarities were also present in the association profile with amino acids (Fig. 2C). Galectin-1 was associated with isoleucine, alanine, and phenylalanine levels, while galectin-3 was associated with leucine and isoleucine and presented an inverse association with tyrosine.

### Metabolon metabolomics and MetaboAnalyst results for galectin-1 and galectin-3

Galectin-1 presented a larger number of statistically significant associations with other metabolites than galectin-3 after adjustment for multiple comparisons (81 versus 19 associated metabolites). Galectin-1-associated metabolites were overrepresented in histidine metabolism, pentose, and glucuronate interconversions, ascorbate and aldarate metabolism, cysteine and methionine metabolism, as well as in the citrate cycle (TCA cycle) (Table 2). Galectin-3 associated metabolites were overrepresented in sphingolipid metabolism and glycosylphosphatidylinositol (GPI)-anchor biosynthesis (Table 2).

**Table 2:** Significant metabolic pathways for galectin-1 and galectin-3 identified using the MetaboAnalyst pathway analysis tool.

Pathway name	*p* value
Galectin-1	
Histidine metabolism	0.0016
Pentose and glucuronate interconversions	0.0023
Ascorbate and aldarate metabolism	0.0061
Cysteine and methionine metabolism	0.013
Citrate cycle (TCA cycle)	0.037
Galectin-3	
Sphingolipid metabolism	5.21*10^−4^
Glycosylphosphatidylinositol (GPI)-anchor biosynthesis	0.027

### Proteomic associations for galectin-1 and galectin-3

Galectin-1 presented a larger number of statistically significant associations with other proteins than galectin-3 after Bonferroni-adjustment for multiple comparisons (354 versus 57 associated proteins). Associated proteins were used to examine associated pathways using the Reactome pathway analysis tool, with several similarities between galectin-1 and galectin-3 regarding the most highly associated pathways (Table 3). Both galectin-1 and galectin-3 were associated with proteins in interleukin and tumor necrosis factor-signaling. Galectin-1 was also associated with the more general pathway of the immune system, as well as neutrophil degranulation. In contrast, galectin-3 was associated with TP53-regulated cell death. Neither galectin presented significant associations with the general pathways of carbohydrate, lipid, or amino acid metabolism. However, both galectin-1 and galectin-3 were significantly associated with proteins in the triglyceride metabolism pathway (*p* = 0.015 and *p* = 0.005, respectively). While plasma galectin-1 and plasma galectin-3 levels were both associated with the proteins fatty acid binding protein (FABP) 4 and -5 in this pathway, only galectin-1 was also associated with FABP1, perilipin-1, and perilipin-3 (Table 4). Plasma levels of both galectins were also associated with the LDL-receptor (*p* < 0.001 for both). Furthermore, plasma galectin-1 but not plasma galectin-3 associated with proteins in the transcriptional regulation of the white adipocyte differentiation pathway (*p* = 0.006 and *p* = 0.100, respectively). Proteins from this pathway associated with galectin-1 were angiopoietin-like 4, perilipin-1, FABP4, leptin, and transforming growth factor (TGF)-β1. Plasma galectin-1 and plasma galectin-3 also presented significant association with each other, and with other galectins (galectin-1 with galectin-7 and -9, and galectin-3 with galectin-4 and -9) (data not shown).

**Table 3:** The five most highly associated protein-signaling pathways for galectin-1 and galectin-3 identified by the REACTOME pathway analysis tool.

Pathway name	*p* value
Galectin-1	
Interleukin-10 signaling	2.08*10^−11^
Tumor necrosis factors bind their physiological receptors	2.25*10^−9^
Immune system	2.94*10^−9^
Neutrophil degranulation	4.47*10^−9^
TNFR2 non-canonical NF-kB pathway	1.15*10^−7^
Galectin-3	
Interleukin-10 signaling	1.24*10^−8^
TP53 regulates transcription of cell death genes	6.02*10^−5^
TP53 regulates transcription of death receptors and ligands	1.04*10^−4^
Tumor necrosis factors bind their physiological receptors	4.63*10^−4^
Interleukin-4 and Interleukin-13 signaling	6.27*10^−4^

**Table 4:** Proteins associated with galectin-1 and galectin-3 levels from REACTOME-identified metabolic pathways.

Galectin-1	Beta (95% CI)	Galectin-3	Beta (95% CI)
Protein		Protein	
Lipid metabolism			
Fatty acid binding protein 4	0.56 (0.47; 0.66)	Fatty acid binding protein 4	0.27 (0.16; 0.39)
Fatty acid binding protein 5	0.34 (0.26; 0.42)	Fatty acid binding protein 5	0.23 (0.13; 0.32)
Low-density lipoprotein receptor	0.23 (0.14; 0.32)	Low-density lipoprotein receptor	0.23 (0.13; 0.33)
Acyl-CoA-binding protein	0.46 (0.38; 0.54)	Diazepam binding inhibitor, acyl-CoA binding protein	0.26 (0.16; 0.35)
Fatty Acid Synthase	0.19 (0.10; 0.27)	Glycoprotein hormones, alpha chain	0.24 (0.13; 0.34)
Thioredoxin reductase 1	0.19 (0.11; 0.27)		
Fatty acid binding protein 1	0.24 (0.16; 0.32)		
Phosphoethanolamine/phosphocholine phosphatase 1	0.22 (0.14; 0.29)		
Lamin B receptor	0.26 (0.17; 0.34)		
Prostaglandin D2 synthase	0.36 (0.28; 0.43)		
Sulfotransferase 2A1	0.17 (0.09; 0.25)		
Angiopoietin-like 4	0.21 (0.13; 0.29)		
TNF receptor superfamily member 21	0.3 (0.22; 0.37)		
Perilipin 3	0.27 (0.19; 0.34)		
Perilipin 1	0.23 (0.14; 0.31)		
Repulsive guidance molecule BMP co-receptor b	0.36 (0.28; 0.44)		
Carbohydrate metabolism			
Beta-1,4-galactosyltransferase 1	0.21 (0.13; 0.29)	Beta-1,4-glucuronyltransferase 1	0.21 (0.12; 0.30)
Syndecan 1	0.18 (0.1; 0.27)		
Glypican 1	0.20 (0.12; 0.28)		
Osteoglycin	0.39 (0.31; 0.47)		
Brevican	0.22 (0.13; 0.30)		
Agrin	0.31 (0.23; 0.39)		
Ribokinase	0.21 (0.12; 0.30)		
Heparan sulfate proteoglycan 2	0.47 (0.4; 0.54)		
Amino acid metabolism			
Proteasome activator complex subunit 2	0.19 (0.11; 0.28)	Glycoprotein hormones, alpha chain	0.24 (0.13; 0.34)
Thioredoxin reductase 1	0.19 (0.11; 0.27)		
Quinoid dihydropteridine reductase	0.26 (0.17; 0.35)		
Agrin	0.31 (0.23; 0.39)		
Histamine N-methyltransferase	0.33 (0.25; 0.41)		
Transcriptional regulation of white adipocyte differentiation			
Fatty acid binding protein 4	0.46 (0.38; 0.54)	Fatty acid binding protein 4	0.27 (0.16; 0.39)
Angiopoietin-like 4	0.21 (0.13; 0.29)		
Perilipin 1	0.23 (0.14; 0.31)		
Leptin	0.43 (0.28; 0.57)		
Transforming growth factor beta 1	0.22 (0.14; 0.29)		

*CI* confidence interval.

## Discussion

We thoroughly characterized the association patterns of galectin-1 and galectin-3 to established and exploratory markers of metabolic disease in a community-based cohort. This allowed us to identify clear distinctions and striking similarities between the two blood-based biomarkers in different aspects of metabolism. While plasma galectin-1 was associated with adipose tissue markers on both an anatomic and proteomic level, galectin-3 showed no such tendency. Conversely, the two galectins were sometimes associated with different insulin sensitivity markers. Galectin-1 was associated with C-peptide, TyG-index and the Matsuda index, while galectin-3 was associated with fasting insulin and TyG-index in the fully adjusted models. Both presented negative associations with fasting glucose, and positive associations with cholesterol, fatty acid, and triglyceride metabolism. Similar associations on the protein level reflected this, with associated proteins including FABP4 and -5, as well as the LDL-receptor. A potential metabolic role of these galectins is interesting, as galectin inhibitors are studied in clinical trials [1].

There was a clear distinction between the two galectins when comparing the association profiles and the Imiomics analysis for galectin-1 and galectin-3 on measures of obesity and adipose tissue distribution. Plasma levels of galectin-1 were closely associated with all adiposity variables, both in the subcutis and viscera. Ectopic fat deposition in the liver and pancreas were no longer significant after BMI adjustment, suggesting indirect associations secondary to BMI. Several studies have reported close associations between galectin-1 and adipose tissue measures [13, 14, 37], and studies in animal models suggest a role for galectin-1 in adipocyte handling of lipids [16, 17]. Several previously proposed mechanisms fit with the observations we find in the proteomic analysis, suggesting that galectin-1 may play a role in adipose tissue organization also in humans [17, 38]. It could be speculated that galectin-1 interacts with leptin, FABP1, -4 and -5, TGF-β1, and perilipin-1 and -3 to modulate whole body lipid storage as indicated by our measurements on BMI, body fat, and Imiomics. The absence of associations between circulating galectin-3 levels and adipose tissue deposition was unexpected, given that both galectins were associated with plasma levels of LDL-cholesterol, triglycerides and fatty acids as well as protein markers of lipid metabolism including the LDL-receptor, FABP4 and -5. However, the observational design does not allow for any conclusion regarding function of the galectins, and it is possible that these associations are mediated by unknown factors. It could also be that galectin-3 has a more prominent role in other metabolically active organs than the adipose tissue, such as the liver. This idea is currently explored as pharmacological galectin-3 inhibitors are under evaluation in clinical trials for the treatment of non-alcoholic steatohepatitis [39].

Several reports have previously indicated associations for galectin-1 with glucose and insulin, and a functional role is suggested in animal models [13, 14, 17]. Here, galectin-1 was associated with all glucose homeostasis variables except for end-OGTT glucose value before BMI adjustment. The closest associations were seen for C-peptide and the insulin resistance measures, HOMA, TyG-index and the Matsuda index, suggesting a role involving insulin resistance rather than glucose or insulin itself. As these associations were mitigated or lost after adjustments for BMI, the functional role of galectin-1 may lie in adipose tissue metabolism as suggested previously [16, 17]. The association with C-peptide aligns with a proposed role of galectin-1 on insulin release in the pancreas of mice [40]. On the contrary, plasma galectin-3 was not associated with C-peptide and pancreas function. However, in similarity with galectin-1, it was associated with TyG index, a marker of insulin resistance that is superior to HOMA-index in Nonalcoholic Fatty Liver Disease (NAFLD) [41]. Galectin-1 has previously been associated with an improved glucose uptake, independent of insulin secretion, which may explain the inverse association observed for both galectins with fasting glucose [42, 43]. This could also explain the lack of association with the 2-h glucose measurement. Fasting glucose levels are closely dependent on hepatic glucose production, regulated by the fasting insulin levels. Thus, a direct effect of the galectins on the hepatocyte is another possibility for the inverse association. Plasma galectin-3 was associated with TyG-index, but not with any other marker of insulin resistance. Several studies have previously found associations between galectin-3 and insulin resistance markers in experimental studies in animal models [10, 44]. The different associations for plasma galectin-1 and -3 with the various markers of insulin resistance may reflect involvement in different metabolic contexts, which should be validated in mechanistic studies [45].

Several proposed ligands are shared between galectin-1 and galectin-3, independently identified in separate studies [46, 47]. It has been argued that these interactions mediate cellular responses, although this remains to be definitively shown [5]. Our study measuring both galectins in the same cohort found similar association patterns between them and several lipid metabolites, as well as markers of lipid metabolism and inflammation, suggestively indicating an overlapping role for the two galectins in these processes. The similar associations with proteins in triglyceride metabolism and the LDL-receptor further support this. In spite of overlapping interaction profiles in vivo and in vitro [48], the global expression is somewhat different. Galectin-1 protein expression is highest in adipose tissue, muscle, and tissues present in females, while galectin-3 expression is highest in the gastrointestinal tract, lungs, skin, kidneys, and bone marrow (https://www.proteinatlas.org/, last accessed on 2022-02-15).

In the targeted metabolomics analysis, associations with cholesterol, fatty acid, and triglyceride markers were almost identical, with few exceptions including free-cholesterol and HDL-metabolites, where statistical significance was not matching. This lends further support for the possibility of overlapping functionalities between the two galectins. It was interesting to find similar associations with both LDL cholesterol and triglycerides for the galectins, as BMI is normally more closely associated with TG than with LDL. Nonetheless, both LDL and triglycerides are known to associate with abdominal fat deposition and a positive energy balance [49, 50]. This aligns with previous studies in galectin-1 deficient mice, and experiments using a galectin-3 specific inhibitor, which have reported that galectin-1 and galectin-3 may interact with the PPAR-γ pathway although in different tissues [11, 16]. Additionally, both galectin-1 and galectin-3 were associated with IL-10 and TNF signaling, pathways previously related to galectins [51–54] and with a role in obesity-related inflammation and lipid metabolism [55, 56].

In our non-targeted metabolomics analysis, the two galectins presented associations with metabolites in distinctly different pathways. Galectin-1-associated metabolites were related to histidine and cysteine metabolism, which both have a known role in galectin-1 function [57, 58]. Pentose and glucuronate interconversion and the TCA cycle pathways are related to carbohydrate metabolism, which may tie together with our observed associations with clinical variables. Pentose and glucuronate interconversions, histidine metabolism, and ascorbate and aldarate metabolism have previously been reported to be altered together in other inflammatory contexts [59, 60]. If galectin-1 and inflammation is linked through these metabolic pathways remains to be determined in future studies. Galectin-3 was related to sphingolipid metabolism, which may be explained by the reported capacity for galectin-3 to interact with glycosphingolipids, e.g., during endocytosis [61]. Sphingolipid metabolism and GPI-anchor biosynthesis pathways have previously been reported to be altered together in a study of a lipid-lowering drug on high-fat-fed mice [62]. These metabolic pathways may be related to our other observed associations between galectin-3 and lipid metabolism, including fatty acids, cholesterols, and FABP4 and -5.

## Strengths and limitations

This study provides new data on how plasma galectin-1 and galectin-3 associate with variables from a comprehensive metabolic characterization in a population-based study. The combination of several different “omic” techniques, with measurements of both galectin-1 and galectin-3 within the same study, allows for a better understanding of similarities and differences between the two lectins on a whole-body level. There are also some limitations to consider in this study. The POEM cohort consists almost exclusively of white participants, and may not be generalizable to other ethnicities. The cross-sectional design does not allow for any conclusions on the direct contribution of galectin-1 and -3 to the studied profiles. We only stratified the Imiomics by sex, because of the sample size. However, the stratified analysis did not indicate any major difference between males and females, and all analyses were adjusted for sex to limit potential bias. The association between galectin-1 and adiposity also complicates the analysis of other variables related to obesity, as there is a risk in over-adjusting the linear models. As galectin-1 can be an agent in obesity released from the adipocytes to regulate metabolic actions, adjusting for BMI might introduce bias to the analysis. Therefore, we present associations with clinical variables before and after adjustment for BMI.

## Conclusion

Taken together, we show that while galectin-1 and galectin-3 in plasma reveal distinctly different associations with obesity and adipose tissue distribution, they also present very similar associations with markers of glucose and lipid metabolism, including cholesterol, fatty acids, and triglycerides. Thus, we find that plasma galectin-1 and galectin-3 have overlapping metabolic associations but profiled toward different tissues. Functional studies are warranted to investigate the metabolic role of galectin-1 and galectin-3 in vitro and in vivo for validation of these results. This information may reveal if galectins hold future potential for treatment of metabolic diseases.

### Supplementary information

Supplementary figure

Supplementary table

## Data Availability

The datasets analyzed during the current study are not publicly available due to restrictions in the ethical permission, but the data can be accessed through the corresponding author upon reasonable request and with permission of the POEM study steering committee.
