# Supplementary material for: Metabolic profiling of galectin-1 and galectin-3: a cross-sectional, multi-omics, association study
Source: Int J Obes (Lond). 2024 May 22;48(8):1180–9. doi: 10.1038/s41366-024-01543-1 (PMC11281902; doi:10.1038/s41366-024-01543-1)
Supplement: Supplementary file 1 — Supplementary figure [file 41366_2024_1543_MOESM1_ESM.docx]

**Supplementary figure**


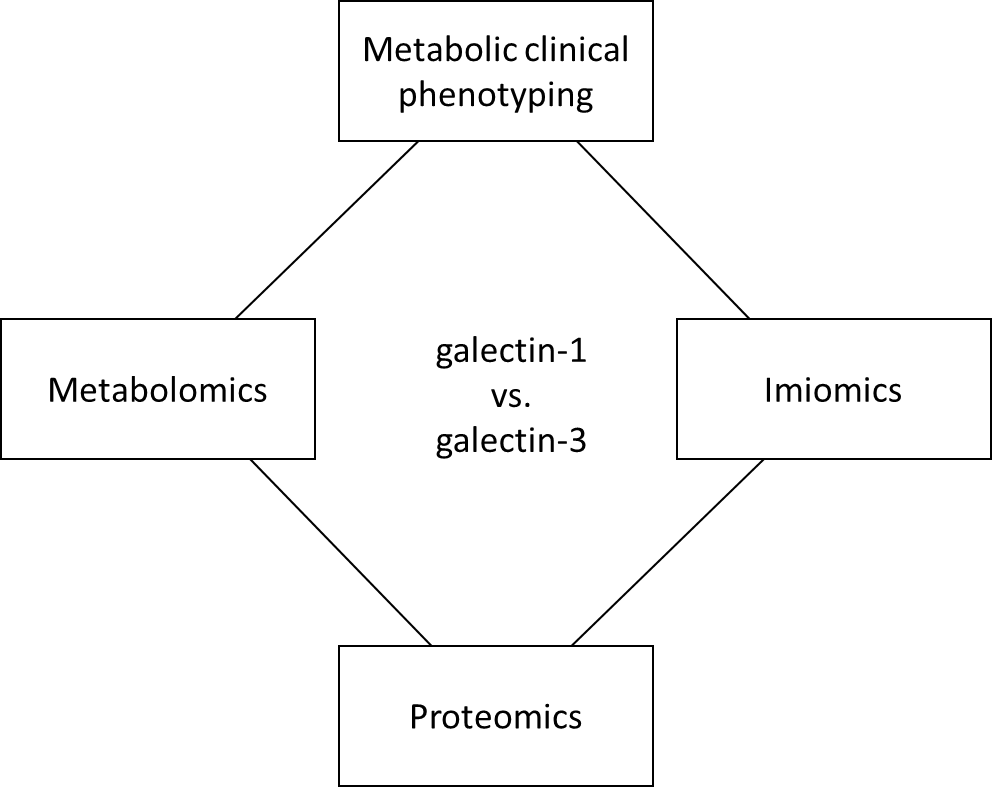


**Supplementary Figure.** Study design: This study adopts a cross-sectional, population-based association study design in a cohort of mainly white participants from urban Uppsala, Sweden. Individuals from the community-based POEM cohort study were characterized by clinical variables of body composition and glucose metabolism. Metabolomics and proteomics were performed on blood, and Imiomics were performed using whole-body magnetic resonance imaging examinations. Galectin-1 and galectin-3 levels were measured in blood and associated with all measured variables in linear regression models adjusted for sex, education, smoking, physical activity, and body mass index.
