# Supplementary material for: Metabolic profiling of galectin-1 and galectin-3: a cross-sectional, multi-omics, association study
Source: Int J Obes (Lond). 2024 May 22;48(8):1180–9. doi: 10.1038/s41366-024-01543-1 (PMC11281902; doi:10.1038/s41366-024-01543-1)
Supplement: Supplementary file 2 — Supplementary table [file 41366_2024_1543_MOESM2_ESM.docx]

| **Supplementary Table.** Characteristics of the POEM cohort. | |
| --- | --- |
| n | 502 |
| Age | 50 (0.1) |
| Female sex (%) | 50 |
| BMI (kg/m^2^) | 26.4 (4.2) |
| Waist circumference (cm) | 92 (11) |
| HDL-cholesterol (mmol/L) | 1.3 (0.3) |
| Triglycerides (mmol/L) | 1.2 (0.9) |
| Fasting insulin (mU/L) | 6.6 (10.6) Median 3.6 |
| Fasting glucose (mmol/L) | 4.9 (0.9) |
| Systolic blood pressure (mmHg) | 126 (16) |
| Diastolic blood pressure (mmHg) | 77 (10) |
|  |  |
| *Total fat (%)* |  |
| All | 28 (8.0) |
| Men | 22 (5.2) |
| Women | 33 (7.2) |
|  |  |
| *Fat distribution (trunk/leg fat mass ratio)* |  |
| All | 1.00 (0.25) |
| Men | 1.20 (0.17) |
| Women | 0.81 (0.15) |
|  |  |
| Smokers (%) | 9.8 |
| Alcohol intake | NA |
| Exercise habits | 2.8 (1.01) (4-grade scale) |
|  |  |
| *Education (%)* |  |
| <10 years | 8 |
| 10-12 years | 44 |
| >12 years | 48 |
|  |  |
| Data presented as means and standard deviations (SD) or proportions (%) |  |
| Abbreviations: BMI, body mass index; HDL, high density lipoprotein; NA, not assessed. Adapted from references 19 and 20. |  |
